# Translation, cross-cultural adaptation, and validation of the Chinese version of the injury-psychological readiness to return to sport scale

**DOI:** 10.1186/s13102-025-01127-0

**Published:** 2025-04-09

**Authors:** Siqi Liu, Yunxi Zhang, Young-Eun Noh, Tong Zhou

**Affiliations:** 1 Faculty of Sports and Exercise Science, Universiti Malaya, Kuala Lumpur, Malaysia; 2 School of Vocational Education, Xi’an Eurasia University, Xi’an, 710065 Shaanxi China; 3 Department of Physical Education, Korea University, Seoul, Republic of Korea

**Keywords:** Sport injury, Return to competition, Validity, Chinese athlete, Psychological barrier

## Abstract

**Background:**

The Injury-Psychological Readiness to Return to Sport (I-PRRS) scale has demonstrated acceptable reliability and validity in American English, Dutch, Italian, Persian, French, Spanish, and Portuguese, however, its adaptation to the Chinese remains unexplored. Therefore, this study aimed to translate the I-PRRS scale into Chinese (I-PRRS-Ch) and validate its cross-cultural adaptation.

**Methods:**

This study employed a cross-cultural adaptation and psychometric testing design to translate and validate the Chinese version of the I-PRRS (I-PRRS-Ch). The translation process followed established guidelines, including forward and backward translation by bilingual experts, followed by an expert panel review to ensure content validity. A pilot study was conducted to assess face validity and identify any potential translation or cultural adaptation issues. The floor and ceiling effects, test-retest reliability, composite reliability, convergent validity, and concurrent validity were assessed to evaluate the translated questionnaire’s reliability and validity. To evaluate the reliability of the I-PRRS-Ch, test-retest reliability was employed using the intraclass correlation coefficient (ICC 3,1). Composite reliability and convergent validity were assessed using partial least squares structural equation modeling (PLS-SEM). The Chinese version of the Tampa Scale of Kinesiophobia (SC-TSK) was used to measure the concurrent validity of the I-PRRS-Ch.

**Results:**

A total of 183 injured athletes (male: *n* = 148, female: *n* = 35; age: *Mean* = 20.04, *SD* = 3) from various sports, including track and field, football, basketball, martial arts, volleyball, and gymnastics, participated in this study. Preliminary analysis showed no floor or ceiling effects were detected for the I-PRRS-Ch. Test-retest reliability of the I-PRRS-Ch scale was excellent (ICC = 0.98). Internal consistency measures included a Cronbach’s alpha value of 0.85 and a composite reliability of 0.89, indicating good reliability. Convergent validity was established with an average variance extracted of 0.57. Concurrent validity was supported by a moderate inverse correlation (*r* = −.42) between the I-PRRS-Ch scale and the SC-TSK, validating the psychological readiness measure concerning kinesiophobia.

**Conclusions:**

The I-PRRS-Ch scale is a reliable and valid tool that provides a screening mechanism to identify psychological barriers in Chinese athletes before returning to sport.

**Supplementary Information:**

The online version contains supplementary material available at 10.1186/s13102-025-01127-0.

## Introduction

A systematic review found that 64.7% of the 795 injured athletes who could not return to sports (RTS) attributed their inability to do so to psychological reasons [1]. Fear of reinjury [2], lack of confidence [3], and low motivation [4] are among the psychological barriers consistently reported by injured athletes. Therefore, even if athletes are physically ready to resume playing sports, they might still face psychological hurdles that impede successful RTS outcomes [5]. 

Given the crucial role of psychological readiness in the success rates of RTS [6], the Injury-Psychological Readiness to Return to Sport (I-PRRS) scale [7] was developed as the most reliable, valid, and sport-specific instrument to assess injured athletes’ confidence levels during rehabilitation. Moreover, it is applicable to various types of injuries [7]. The I-PRRS scale has been translated into several languages, including Dutch [8, 9], Italian [10], Persian [11], French [12], Spanish [12], and Portuguese [12]. It has consistently demonstrated strong reliability and validity across various studies and cultural contexts [8–12]. 

However, its adaptation to the Chinese context remains unexplored. Recurring injuries among Chinese athletes [13] highlight the need for a Chinese-language tool to assess the psychological challenges these athletes face in terms of RTS and potentially mitigate injury recurrence. Therefore, this study aims to translate the I-PRRS scale into Chinese (I-PRRS-Ch) and validate its cross-cultural adaptation. The I-PRRS-Ch scale can assist practitioners in monitoring Chinese injured athletes’ confidence levels during rehabilitation and determining their psychological readiness for RTS, thereby reducing the risk of reinjury among these athletes by identifying and addressing psychological issues. We hypothesized that the Chinese version of I-PRRS is valid and reliable for measuring the psychological readiness to RTS in the Chinese population.

## Methods

### Study design

This study employed a cross-cultural adaptation and psychometric testing study to translate and validate the I-PRRS into Chinese (I-PRRS-Ch). The research was conducted in two main phases: Study 1 focused on ensuring content validity through translation and expert review, and Study 2 aimed to assess the reliability and validity of the translated questionnaire. The translation process followed established guidelines [14], including forward and backward translation by bilingual experts, an expert panel review to check content validity, and a pilot study to check face validity and identify any potential translation or cultural adaptation issues. Test-retest reliability, composite reliability, convergent validity, and concurrent validity were assessed to evaluate the translated questionnaire’s reliability and validity.

### Study 1: ensuring content validity in questionnaire translation

The primary aims of Study 1 included translating the I-PRRS scale with the assistance of four interpreters, validating its content through consultation with a panel of content specialists, and evaluating the face validity with 30 athletes.

#### Initial translation

We invited four interpreters to translate the I-PRRS scale into Chinese using the forward-backward translation method [14]. This involved a 5-step process: (1) forward translation; (2) backward translation; (3) an expert panel review for accuracy, cultural relevance, and appropriateness; (4) a pilot survey for identifying any issues with understanding, interpretation, and cultural relevance; and (5) an expert panel review for making changes based on the results of the pilot survey.

Two English-speaking interpreters independently carried out the forward translation into Chinese. Then, two interpreters, unaware of the original (English) version of the scale, independently conducted the backward translation into English. They compared the original English text with the retranslated version for accuracy. Any discrepancies were resolved through discussion. All the translators possessed professional translating experience (5–7 years).

#### The panel of experts: content validity

We invited a panel of experts to assess the content validity [15] of the I-PRRS-Ch scale. To be considered an expert for this study, individuals had to meet at least 1 of the following criteria: (1) hold a doctoral degree in sport psychology and have a track record of publishing articles in internationally recognized journals focusing on RTS after sport injuries, (2) be certified as a psychologist with at least 5 years of relevant working experience, and (3) possess a coaching certification and have a minimum of 5 years of practical coaching experience. Based on these criteria, we formed an expert panel by inviting three individuals with extensive knowledge and experience in their respective fields and asked them to provide insights into and evaluations of the I-PRRS-Ch scale.

#### The pilot study

**Participants**. Individuals were eligible for the pilot survey if they (1) were over 18 years old, (2) had a minimum of 1 year of sport training or competition experience, (3) had sustained sport injuries that resulted in at least 7 days of absence from sport activities, and (4) could proficiently read simplified Chinese, which enabled them to complete the questionnaire either manually or orally. The option for oral completion was included to accommodate injured athletes who may have had physical limitations preventing them from filling out the questionnaire manually. During the pilot survey, 30 athletes with a history of sport-related injuries were invited to complete the unified I-PRRS-Ch scale on the Wenjuanxing platform [16]. 

**Fig. 1:**
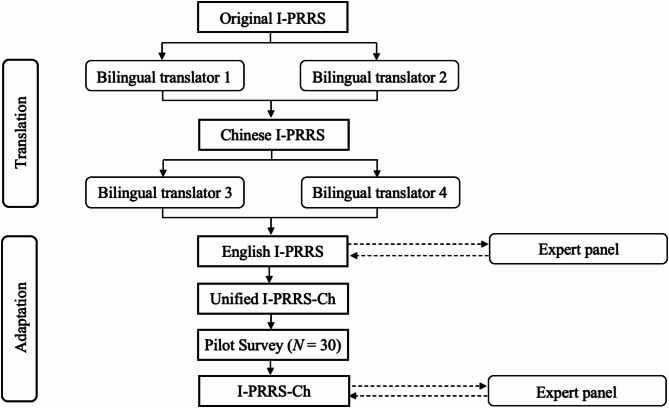
Flowchart of the Translation and Adaptation Procedures for the I-PRRS-Ch Scale. *Note.* I-PRRS = the Injury-Psychological Readiness to Return to Sport scale, I-PRRS-Ch = the Chinese Version of Injury-Psychological Readiness to Return to Sport scale

**Face Validity**. In the next step, the first author interviewed these participants via WeChat, a multi-purpose social media app that is one of the most widely used in China, to assess face validity and identify any potential translation or cultural adaptation issues. The interview questions were based on a previous study [17]. This included evaluating the content of the translated guidelines, the items’ phrasing, and the response options [18]. The questionnaire underwent iterative revisions by the expert panel until the participants confirmed their complete understanding of the guidelines, item content, and answer choices. Figure 1 illustrates the flowchart of the translation and adaptation processes for the I-PRRS-Ch scale.

### Study 2: evaluating the reliability and validity of the translated questionnaire

The objective of Study 2 was to validate the I-PRRS-Ch scale in terms of reliability and validity. We chose the Chinese version of the Tampa Scale for Kinesiophobia (SC-TSK) [19] to assess the concurrent validity of the I-PRRS-Ch scale.

#### Participants

The main study utilized a mixed-mode survey [20], which incorporated both online and offline methods, to collect a broader range of data. This approach was necessary due to our stringent participant-selection criteria and the limited pool of potential respondents. Athletes were eligible to participate if they (1) were over 18 years of age, (2) had at least 1 year of sport training or competition experience with a minimum of 3 practice days per week prior to the injury, (3) had experienced a sport injury resulting in an absence from training or competition of at least 7 days (including any surgical or conservative treatment), (4) were injured and intended to RTS to achieve the same level of performance as before the injury, and (5) were able to read simplified Chinese in order to complete the questionnaire manually or orally. Athletes were excluded if their injuries were not sport-related but were caused, for example, by rheumatic or neurological diseases.

#### Measurement

**Demographic Questionnaire**. This questionnaire gathered demographic information about the participants, including gender, age, training frequency per week, sport history, types of sports practiced, injury history, whether the injury was sport-related, and if the training cessation time exceeded 7 days.

**The Chinese Version of the I-PRRS Scale.** The uni-dimensional I-PRRS scale [7] has 6 items and each item is rated on a scale from 0 (*no confidence at all*) to 10 (*complete confidence*), with the total score ranging from 0 to 60. This total is obtained by summing the scores of each item. A score of 50 or above suggests that the athlete has the psychological readiness necessary for RTS. The Chinese version of the I-PRRS (I-PRRS-Ch) scale revised after the pilot study was utilized to assess its test-retest reliability, composite reliability (CR), convergent validity, and concurrent validity.

**The Chinese Version of the TSK.** [19] The SC-TSK, which was adapted from the 11-item TSK [21], measures fear of movement/(re)injury using a 4-point Likert scale (ranging from 1, *strongly disagree*, to 4, *strongly agree*). It comprises 3 dimensions: somatic focus (items 2, 3, 4, 5, 6, and 8), activity avoidance (items 7, 9, and 10), and avoidance belief (items 1 and 11). The total SC-TSK score can range from 11 to 44, and it reflects the extent of kinesiophobia, with higher scores indicating greater fear. The reliability of the SC-TSK has been established as adequately high (Cronbach’s alpha = 0.883), with excellent test–retest reliability (0.798) [19]. 

#### Procedure

After receiving ethical approval from the University of Malaya Research Ethics Committee (UM.TNC2/ UMREC_2691) and obtaining translation permissions from the developers of the I-PRRS, SC-TSK, and TSK scales, we initiated participant recruitment in China through both offline (e.g., sport clubs, rehabilitation centers, and hospitals) and online channels (e.g., social media). The online survey was distributed via four major Chinese social media platforms: Quick Hand, TikTok, RED, and Sina Weibo through the Wenjuanxing platform [16], a popular online survey and questionnaire platform similar to Google Forms, widely used in China. We targeted Chinese athletes who mentioned on their social media profiles sport-related injuries that prevented their RTS. We sent them a message detailing the study’s purpose, eligibility criteria, and procedures. Interested athletes received preliminary phone calls offering more details and formal invitations. The invitation included the study’s purpose, eligibility criteria, procedures, consent forms, and the link to the questionnaire. All online participants signed the consent forms before providing their demographic information. They then completed the demographic section, the SC-TSK, and I-PRRS-Ch scales. The online system restricted each participant to a single submission to prevent duplicate responses.

We relied on our informal and professional networks to recruit participants offline, including two sports clubs, 13 rehabilitation centers, and three hospitals. We briefed intermediaries (e.g., coaches, physical rehabilitation therapists, and physiotherapists) on the study’s objectives, criteria, and procedures via WeChat. Those who agreed to help distributed paper questionnaires and consent forms to eligible participants. Before completing the questionnaires, the offline participants signed consent forms. The completed questionnaires were collected by the intermediaries. To further assess the reliability of the I-PRRS-Ch scale, we invited 100 participants from the initial survey. These individuals were asked to complete the I-PRRS-Ch scale again within one to two weeks to evaluate its test–retest reliability. This flexible time frame helps alleviate undue pressure on the participants. In the second survey, we included a screening question, “Have you returned to sports?” to ensure that athletes who had resumed sports activities were excluded from the study. During the interim period, no significant events occurred that could influence the participants’ responses, except for three individuals who had returned to sports training.

To ensure compliance with the established procedures, we followed rigorous guidelines throughout the study. To evaluate the reliability and validity of the translated questionnaire, we assessed test-retest reliability, CR, convergent validity, and concurrent validity. Compliance was monitored through regular meetings with the team to ensure adherence to the guidelines. These procedures were consistently followed to enhance the reliability and validity of the study.

#### Data analysis

**Sample Size Calculation.** The minimum sample size for conducting confirmatory factor analysis (CFA) was determined to be 137, based on power analysis conducted with the A-priori Sample Size Calculator for Structural Equation Models [22]. This analysis took into account a model structure with 4 latent variables and 17 observed variables, a medium effect size (ƒ [2]) of 0.3, a significance level of 0.05, and a desired power level of 0.80, which aligns with standard practices in social science research [23]. CFA is commonly used to assess the construct validity of measurement tools [24], ensuring that the items in a questionnaire or scale accurately reflect the underlying theoretical constructs.

For the assessment of test–retest reliability, we calculated the required sample size using G*Power software. We performed an a priori power analysis, aiming for a power of 0.80 and an alpha level of 0.05 and assuming a medium effect size (Pearson’s *r* =.3) for the test–retest reliability coefficient. Based on these parameters, the power analysis indicated that at least 67 participants were necessary to maintain adequate power for detecting significant reliability in the questionnaire scores over time.

**Floor or Ceiling Effects**. To investigate the potential floor or ceiling effects in the I-PRRS-Ch scale, we utilized IBM SPSS Statistics (version 27). Floor and ceiling effects occur when a significant proportion of respondents achieve the lowest (floor) or highest (ceiling) possible scores on a scale. This restricts the ability of the instrument to distinguish between individuals at these extremes, leading to a loss of measurement sensitivity and potential bias in detecting changes over time. A floor or ceiling effect is typically considered present if approximately 15% of respondents score on the scale items at the lowest or highest possible level [25]. 

**Test-Retest Reliability**. We assessed the test–retest reliability of the I-PRRS-Ch scale using the Intraclass Correlation Coefficient (ICC) based on a 2-way random-effects model for single measures (ICC 3,1). This approach accounts for both the random variability among participants and the fixed nature of the measurement methods. An ICC value of 0.75 or above is interpreted as indicating excellent reliability [26]. Values ranging from 0.60 to 0.75 suggest good reliability, while values between 0.40 and 0.59 indicate fair reliability [26]. 

We calculated the standard error of measurement (SEM) and the minimal detectable change (MDC). The SEM quantifies the error inherent in a test score, estimating the variability in a person’s observed scores due to measurement error. It was calculated using the total variance between the first and second measurements and the ICC [27]. The smaller SEM indicates the greater reliability of the measurement. The MDC represents the smallest change in a score that can be interpreted as a real change, beyond the margin of measurement error. It helps determine whether a score change is meaningful or attributable to an error. The MDC was calculated by multiplying the SEM by 1.96 (for 95% confidence) and the square root of 2.

**Composite Reliability and Convergent Validity**. To evaluate the reliability and convergent validity, we employed SmartPLS (version 4). Partial least squares structural equation modeling (PLS-SEM) was chosen for several reasons. First, the translated I-PRRS-Ch questionnaire is a uni-dimensional scale. Given this simplicity in structure, PLS-SEM was a practical choice for validating the scale’s factorial validity. Second, PLS-SEM involves evaluating the measurement model, which is comparable to conducting a CFA. Since the questionnaire is uni-dimensional, CFA was used to confirm that all items measure the same underlying construct. Additionally, CFA helped evaluate the scale’s psychometric properties, including reliability through factor loadings, Cronbach’s alpha, CR, and convergent validity via average variance extracted (AVE). Lastly, PLS-SEM is particularly advantageous in situations with smaller sample sizes, as it does not impose strict requirements on sample size or data distribution [28]. 

**Fig. 2:**
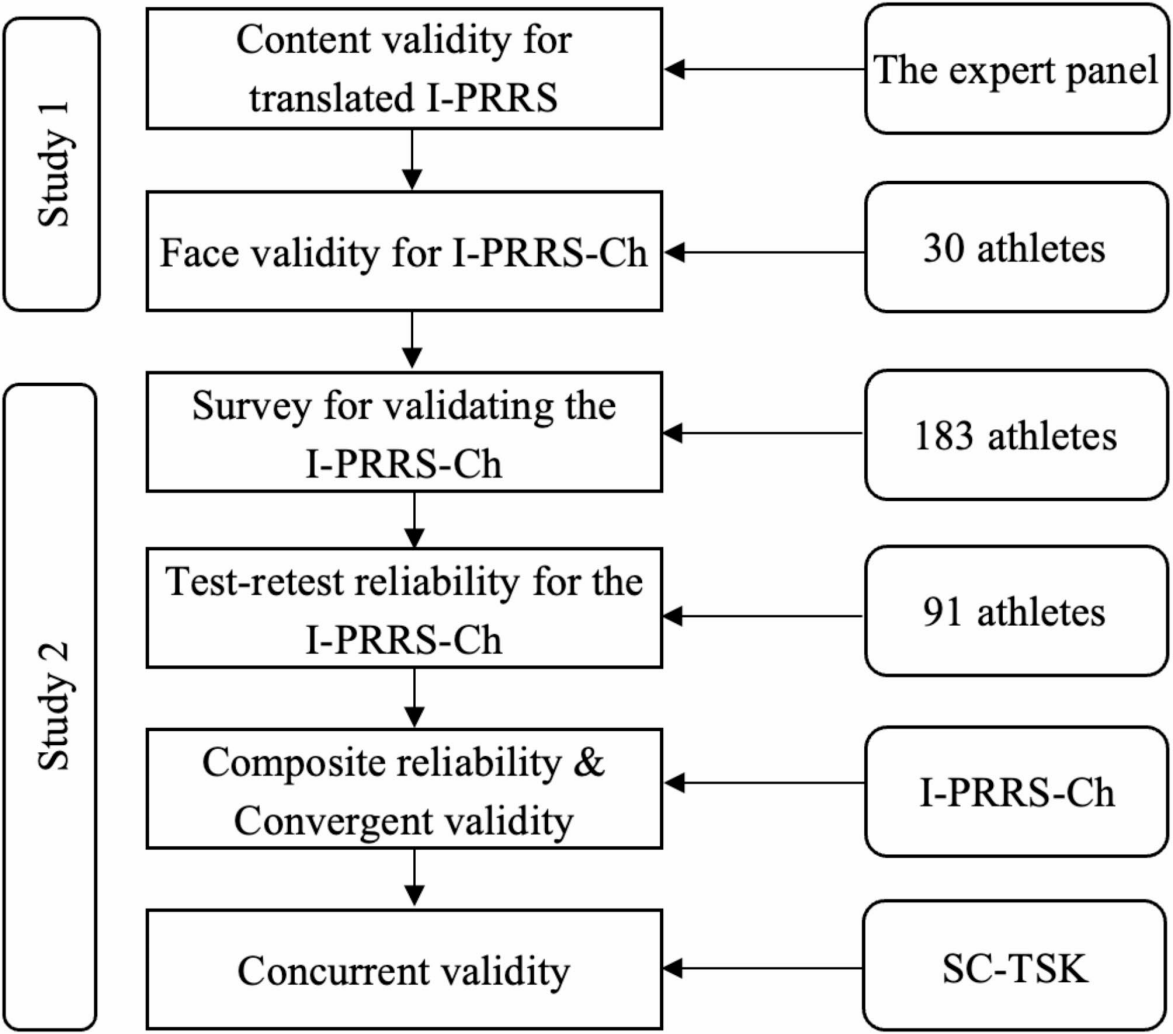
A Flowchart for the Validity and Reliability Process. *Note.* I-PRRS-Ch = the Chinese Version of Injury-Psychological Readiness to Return to Sport scale; SC-TSK = the Chinese version of the Tampa Scale for Kinesiophobia

**Concurrent Validity**. Concurrent validity was evaluated through the calculation of a Pearson product-moment correlation coefficient between the I-PRRS-Ch scale and the SC-TSK. Correlation coefficients exceeding 0.6 were categorized as high, those within the 0.3–0.6 range were classified as moderate, and values below 0.3 were deemed to be low [29]. The flowchart outlining the validity and reliability process is presented in Fig. 2.

## Results

### Content validity

The panel, which includes two sport psychologists with 16 and 28 years of experience and a coach with 15 years of experience, examined the translated version against the original to identify any discrepancies, which were resolved through discussion. It also invited the creator of the I-PRRS scale to review the backward-translated version for accuracy. Following the creator’s suggestions, revisions were made to adapt certain items to the Chinese cultural context. For example, to avoid subjective interpretation, the phrase “100% effort” was retained over “give my best” in the third item. Moreover, for clarity and to prompt a numerical response in the fourth item, the sentence “I won’t be distracted by focusing on my injury” was rephrased as “After returning to sport, my attention will not be diverted by my injury.”

### Face validity

The pilot survey results showed that the respondents could complete the questionnaire in 2–3 min. Table 1 presents the demographics of the pilot study participants.

**Table 1:** Pilot survey participants’ characteristics (*N* = 30)

Demographics	Values	*n*	Percentage
Age	18–20	9	30
	21–23	10	33.3
	24–26	8	26.7
	27–28	3	10
Training frequency	1–2 times/week	7	23.3
	3–4 times/week	15	50
	5–7 times/week	8	26.7
Types of sports	Track and field	9	30
	Football	4	13.3
	Basketball	5	16.7
	Martial arts	3	10
	Volleyball	5	16.7
	Gymnastics	4	13.3
Sport career	1–3 years	5	16.7
	4–6 years	13	43.3
	7–9 years	8	26.7
	≥ 10 years	4	13.3

### Selected participants for the validation of the I-PRRS-Ch

**Fig. 3:**
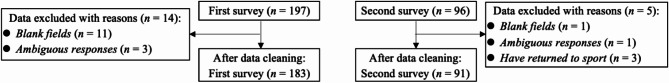
Data Screening Flowchart

During data collection, no participants encountered any issues filling out the questionnaire orally. After data cleaning, we retained 183 responses (male: *n* = 148, female: *n* = 35; age: *Mean* = 20.04, *SD* = 3, range = 18–38) from the first survey and 91 responses from the second survey. The data cleaning flowchart is illustrated in Fig. 3 and the participants’ characteristics for the first and second surveys are shown in Table 2.

**Table 2:** Participants’ characteristics for the first (*N* = 183) and second (*N* = 91) surveys

Demographics	First survey		Secondary survey	
	*n*	Percentage	*n*	Percentage
Data collection method				
Online	52	28.4	21	23.1
Offline	131	71.6	70	76.9
Age				
18–20	131	71.6	72	79.1
21–23	33	18	12	13.2
24–26	7	3.8	3	3.3
27–38	12	6.6	4	4.4
Training frequency				
3–4 times/week	58	31.7	19	20.9
5–6 times/week	80	43.7	41	45.1
≥ 7 times/week	45	24.6	31	34.1
Types of sports				
Track and field	89	48.6	47	51.6
Football	30	16.4	14	15.4
Basketball	5	2.7	2	2.2
Martial arts	6	3.3	2	2.2
Volleyball	28	15.3	11	12.1
Gymnastics	25	13.7	15	16.5
Sport career				
1–3 years	127	69.4	66	72.5
4–6 years	43	23.5	20	22
7–9 years	5	2.7	4	4.4
≥ 10 years	8	4.3	1	1.1

### Floor or ceiling effects

No floor or ceiling effects were detected in either the first or second survey. In the first survey (*N* = 183), the total scores for the I-PRRS-Ch scale ranged from 22 to 60 (*M* = 46.20, *SD* = 9.91). Sixteen injured athletes (8.7%) achieved the maximum score of 60 on the I-PRRS-Ch scale, while no athlete recorded the minimum score of 0. In the second survey (*N* = 91), the total scores varied from 29 to 60 (*M* = 49.15, *SD* = 9.11). Thirteen injured athletes (14.3%) reached the maximum score of 60 on the I-PRRS-Ch scale; this proportion is still below the recommended threshold of 15%.^25^ No injured athlete reported the lowest possible score. The absence of floor or ceiling effects suggests that the I-PRRS-Ch scale effectively captures the full range of variability in psychological readiness levels. Additionally, the scale’s response options and score distribution are well-suited to the Chinese athlete population. The average interval between the two administrations was 10.65 days (*SD* = 2.1).

### Test-retest reliability

For single measures, the ICC was found to be 0.98, with a 95% CI ranging from 0.96 to 0.98, *F*(90, 90) = 78.729, *p* <.001, which indicates excellent reliability. The SEM was 0.28, indicating that the translated I-PRRS has a minimal random error, ensuring high reliability. The MDC was 0.78 points, demonstrating that even small score changes between test and retest can be accurately evaluated, making the scale suitable for research purposes.

### Composite reliability and convergent validity

A comprehensive analysis of the convergent validity of the I-PRRS-Ch scale was conducted, and the results are summarized in Table 3. The factor loadings for all the items fall within a reasonable range (> 0.60), which suggests an acceptable value [30]. The indicators of internal consistency also support the reliability of the scale. The Cronbach’s alpha value of 0.85 and the CR value of 0.89 indicate good internal consistency [31]. The AVE was found to be 0.57, which is above the conventional threshold of 0.50 [28], demonstrating an acceptable level of convergent validity. No items were eliminated after validation, attesting to the overall acceptability of the measurement items.

**Table 3:** Reliability and convergent validity of the I-PRRS-Ch scale

Measurement	Item	Factor loading	Cronbach’s α	CR	AVE
I-PRRS-Ch	B1	0.757	0.852	0.888	0.572
	B2	0.792			
	B3	0.611			
	B4	0.801			
	B5	0.819			
	B6	0.738			

Note. CR = composite reliability; AVE = average variance extracted

### Concurrent validity

There is a moderate inverse correlation between them as indicated by the Pearson product-moment correlation coefficient (*r* = −.42). This significant negative correlation, indicating that higher confidence is associated with lower kinesiophobia, supports the hypothesis and the concurrent validity of the I-PRRS-Ch.

## Discussion

This study aimed to translate the English version of the I-PRRS scale into Chinese, thus creating the I-PRRS-Ch scale, and validating it among Chinese injured athletes. The study found that the I-PRRS-Ch scale possesses adequate psychometric properties, which are comparable to those of the original English scale [7]. Furthermore, both the I-PRRS-Ch scale and the SC-TSK exhibited robust validity indices, which underscores their validity.

In previous studies [8, 11], the I-PRRS has demonstrated excellent test–retest reliability, which indicates significant stability over time across different populations. When applied to a Dutch sample, the scale achieved an intraclass correlation coefficient (ICC) of 0.89, with a 95% confidence interval (CI) ranging from 0.84 to 0.92 [8]. Similarly, when used among Persian-speaking participants, it exhibited outstanding test–retest reliability, with an ICC single measure of 0.97 (95% CI = 0.93–0.98) (*p* <.001) [11].

Regarding validity, the scale has demonstrated strong construct validity—notably, a significant correlation of 0.79 with the ACL-RSI scale [8]—confirming its accuracy in concurrent measurement. Furthermore, CFA has provided evidence of the construct validity of the I-PRRS scale [8–12]. For instance, with an Italian sample, the scale obtained goodness-of-fit values that ranged from 0.64 to 0.90 [10], which suggests a well-fitting model. Likewise, among Persian-speaking respondents, the goodness-of-fit metrics varied from 0.60 to 0.87 [11], which also confirms the scale’s robustness. Similar to the English version of the I-PRRS scale, which has demonstrated acceptable reliability and validity among American [7], Dutch [8, 9], French [12], Spanish [12], and Portuguese athletes [12], the I-PRRS-Ch scale also demonstrated good reliability and validity.

As hypothesized, the negative correlation observed between the I-PRRS-Ch and the SC-TSK aligns with theoretical expectations. The I-PRRS-Ch measures psychological readiness, which includes confidence and a positive mindset about returning to sport [7], while the SC-TSK assesses fear of reinjury and avoidance behaviors associated with kinesiophobia [19]. The previous research suggests a potential link between the degree of kinesiophobia and an athlete’s psychological readiness for RTS [8, 9]. The negative relationship between the I-PRRS-Ch and the SC-TSK suggests that higher psychological readiness is associated with lower levels of kinesiophobia. Athletes who feel more prepared and confident about returning to sport are less likely to experience fear and avoidance behavior, which are detrimental to rehabilitation and performance [32]. Conversely, higher kinesiophobia may hinder psychological readiness due to the athlete’s apprehension about reinjury [32]. 

In this study, the structure of the I-PRRS-Ch scale aligns with the English [7] and Dutch versions [8], retaining a 1-factor structure with 6 items, as well as with the French [12], Spanish [12], and Portuguese versions [12]. In contrast, the Italian [10] and Persian versions [11] underwent modifications after translation and validation within their respective cultural contexts, resulting in 2-factor structures. Specifically, the Italian version [10] features 2 subscales, namely “Confidence in Performance Capability” (items 1, 3, and 5) and “Confidence in Recovery” (items 2, 4, and 6). Similarly, the Persian version [11] is divided into “Confidence to Play” (items 1 and 2) and “Confidence in the Injured Body Part and Skill Level” (items 3, 4, 5, and 6). These variations reveal the influence of cultural factors on the structure of measurement instruments and highlight the importance of studying cross-cultural adaptation in future research.

The cross-cultural validation of the I-PRRS scale is particularly relevant given the growing interest in understanding the cognitive and emotional responses of injured athletes. Performing such validation can offer reliable screening tools for athletes from diverse cultural and linguistic backgrounds, facilitating their RTS. To the best of our knowledge, no previous study has validated the adaptation of the I-PRRS scale with a sample of Chinese injured athletes. Our work also encourages further localization of questionnaires designed to assess the psychological readiness of Chinese athletes following sport injuries.

One point to note is that the participants in this study had relatively short athletic careers (1–3 years), which may be associated with a higher likelihood of injury among novice athletes. Li et al. (2023) found that young athletes in China who are new to professional sports may be more prone to injuries due to underdeveloped technical skills or excessive training loads [33]. In contrast, elite athletes with multiple previous injuries may exhibit different levels of psychological readiness to RTS following injury [34]. Therefore, future research could benefit from including a broader sample of athletes with varying age ranges, sports experience, and injury histories to better understand these dynamics.

### Study limitations and future research

This study has significant limitations. Firstly, the study did not consider factors such as the type of injury, which could affect athletes’ return to competitive activity [35]. Secondly, the use of a mixed-mode survey (online and offline surveys) might affect the validity of the I-PRRS-Ch. For example, participants may respond differently to questions depending on the mode of data collection. Online responses might be more candid due to perceived anonymity, while offline responses might be influenced by social desirability bias. Thirdly, although no participants in this study completed the scale orally, administering surveys both orally and in written form could introduce bias in face validity due to differences in how respondents perceive and interpret the items. These differences in interpretation may lead to inconsistencies in how the questionnaire is perceived, potentially affecting its apparent relevance and clarity. Lastly, the study cohort predominantly consisted of male participants, which may limit the generalizability of the findings to female athletes. In the future scholars should aim to recruit larger cohorts of injured athletes to establish normative data and categorize the readiness for RTS by considering variables such as competition level, injury type, rehabilitation duration, and history of previous injuries.

## Conclusion

The I-PRRS-Ch scale is a valid and reliable tool for use among Chinese-speaking populations. It can effectively serve as a screening tool to identify potential psychological barriers in Chinese athletes prior to RTS.

## Electronic supplementary material

Below is the link to the electronic supplementary material.

Supplementary Material 1

## Data Availability

All data generated and/or analyzed during this study are available from the corresponding author upon reasonable request.

## References

[1] Nwachukwu BU, Adjei J, Rauck RC, et al. How much do psychological factors affect lack of return to play after anterior cruciate ligament reconstruction? A systematic review. Orthop J Sports Med. 2019;7:2325967119845313. 31205965 10.1177/2325967119845313 PMC6537068

[2] DiSanti J, Lisee C, Erickson K, et al. Perceptions of rehabilitation and return to sport among high school athletes with anterior cruciate ligament reconstruction: a qualitative research study. J Orthop Sports Phys Ther. 2018;48:951–9. 29932875 10.2519/jospt.2018.8277

[3] O’Connor S, Moran K, Sheridan A, et al. Fear avoidance after injury and readiness to return to sport in collegiate male and female Gaelic games players. Sports Health. 2021;13:532–9. 33682535 10.1177/1941738121999047 PMC8558997

[4] Trainor LR, Crocker PRE, Bundon A, et al. The rebalancing act: injured varsity women athletes’ experiences of global and sport psychological well-being. Psychol Sport Exerc. 2020;49:101713.

[5] Seil R, Mouton C, Lion A, et al. There is no such thing like a single ACL injury: profiles of ACL-injured patients. Orthop Traumatol Surg Res. 2016;102:105–10. 26776099 10.1016/j.otsr.2015.11.007

[6] Gokeler A, Dingenen B, Mouton C, Seil R. Clinical course and recommendations for patients after anterior cruciate ligament injury and subsequent reconstruction: a narrative review. EFORT Open Rev. 2017;2:410–20. 29209517 10.1302/2058-5241.2.170011 PMC5702954

[7] Glazer DD. Development and preliminary validation of the injury-psychological readiness to return to sport (I-PRRS) scale. J Athl Train. 2009;44:185–9. 19295964 10.4085/1062-6050-44.2.185 PMC2657021

[8] Slagers AJ, Reininga IHF, Geertzen JHB, Zwerver J, van den Akker-Scheek I. Translation, cross-cultural adaptation, validity, reliability and stability of the Dutch injury-psychological readiness to return to sport (I-PRRS-NL) scale. J Sports Sci. 2019;37:1038–45. 30394202 10.1080/02640414.2018.1540101

[9] Vereijken A, Aerts I, van Trijffel E, Meeusen R. Translation and validation of the Dutch injury psychological readiness to return to sport scale (I-PRRS). Int J Sports Phys Ther. 2019;14:785–93. 31598416 PMC6769267

[10] Conti C, di Fronso S, Robazza C, Bertollo M. The injury-psychological readiness to return to sport (I-PRRS) scale and the sport confidence inventory (SCI): a cross-cultural validation. Phys Ther Sport. 2019;40:218–24. 31610419 10.1016/j.ptsp.2019.10.001

[11] Naghdi S, Ansari NN, Farhadi Y, et al. Cross-cultural adaptation and validation of the injury-psychological readiness to return to sport scale to Persian Language. Physiother Theory Pract. 2016;32:528–35. 27618418 10.1080/09593985.2016.1221486

[12] Dunlop G, Ivarsson A, Andersen TE, et al. Examination of the validity of the Injury-Psychological readiness to return to sport (I-PRRS) scale in male professional football players: a worldwide study of 29 professional teams. J Sports Sci. 2023;41:1906–14. 38269550 10.1080/02640414.2024.2307764

[13] Li Y, Schinke RJ, Middleton TRF, et al. The contextualisation of Chinese athletes’ careers in the Chinese whole Nation system. Int J Sport Exerc Psychol. 2023;21:138–55.

[14] Beaton DE, Bombardier C, Guillemin F, Ferraz MB. Guidelines for the process of cross-cultural adaptation of self-report measures. Spine. 2000;25:3186–91. 11124735 10.1097/00007632-200012150-00014

[15] Mokkink LB, Terwee CB, Knol DL, et al. The COSMIN checklist for evaluating the methodological quality of studies on measurement properties: a clarification of its content. BMC Med Res Methodol. 2010;10:1–8. 20298572 10.1186/1471-2288-10-22 PMC2848183

[16] Wenjuanxing. A survey for validity, reliability and stability of the chinese injury-psychological readiness to return to sport (I-PRRS-Ch) scale. February 25, 2004. Accessed July 28, 2023. https://www.wjx.cn/

[17] Liu S, Noh Y. Beyond physical recovery: investigating athletic identity as a mediator between social support and psychological readiness for return to sport. Aust J Psycholy. 2024;76:2402424. 10.1080/00049530.2024.2402424 PMC12218440 40666658

[18] Taherdoost H. Validity and reliability of the research instrument; how to test the validation of a questionnaire/survey in a research. Int J Acad Res Manag. 2016;5:28–36.

[19] Cai L, Liu, et al. Cross-cultural adaptation, reliability, and validity of the Chinese version of the Tampa scale for Kinesiophobia-11 among patients who have undergone total knee arthroplasty. J Arthroplasty. 2019;34:1116–21. 10.1016/j.arth.2019.01.076 30853160

[20] Dillman DA, Smyth JD, Christian LM. Internet, phone, mail, and Mixed-Mode surveys: the tailored design method. 4th ed. Wiley; 2014.

[21] Woby SR, Roach NK, Urmston M, Watson PJ. Psychometric properties of the TSK-11: a shortened version of the Tampa scale for kinesiophobia. Pain. 2005;117:137–44. 16055269 10.1016/j.pain.2005.05.029

[22] Soper [Computer Software]. A-priori sample size calculator for structural equation models; Daniel Soper; 2023. https://www.danielsoper.com/statcalc/calculator.aspx?id=89

[23] Hair JF, Astrachan CB, Moisescu OI, et al. Executing and interpreting applications of PLS-SEM: updates for family business researchers. J Fam Bus Strat. 2021;12:100392.

[24] Tavakol M, Wetzel A. Factor analysis: a means for theory and instrument development in support of construct validity. Int J Med Educ. 2020;11:245–47. 33170146 10.5116/ijme.5f96.0f4a PMC7883798

[25] Terwee CB, Bot SD, de Boer MR, et al. Quality criteria were proposed for measurement properties of health status questionnaires. J Clin Epidemiol. 2007;60:34–42. 17161752 10.1016/j.jclinepi.2006.03.012

[26] Fleiss JL, Levin B, Paik MC. Statistical methods for rates and proportions. Wiley; 2013.

[27] de Vet HCW, Terwee CB, Knol DL, Bouter LM. When to use agreement versus reliability measures. J Clin Epidemiol. 2006;59:1033–9. 16980142 10.1016/j.jclinepi.2005.10.015

[28] Hair JF Jr, Hult GTM, Ringle CM, Sarstedt M. A primer on partial least squares structural equation modeling (PLS-SEM). 2nd ed. Sage; 2021.

[29] Hinkle DE, Wiersma W, Jurs SG. Applied statistics for the behavioral sciences. Houghton MiZin; 1998.

[30] Chin WW. The partial least squares approach to structural equation modeling. In: Marcoulides GA, editor *Modern methods for business research*. Lawrence Erlbaum Associates;1998:295–336.

[31] Hair JF, Black WC, Babin BJ, Anderson RE. Multivariate data analysis. 7th ed. Prentice Hall; 2010.

[32] Mahood C, Perry M, Gallagher P, Sole G. Chaos and confusion with confidence: managing fear of Re-Injury after anterior cruciate ligament reconstruction. Phys Ther Sport. 2020;45:145–54. 32777712 10.1016/j.ptsp.2020.07.002

[33] Li Y, Schinke RJ, Middleton TRF, Li P, Si G, Zhang L. The contextualisation of Chinese athletes’ careers in the Chinese whole Nation system. Int J Sport Exerc Psychol. 2023;21:138–55.

[34] Zarzycki R, Cummer K, Arhos E, et al. Female athletes with better psychological readiness are at higher risk for second ACL injury after primary ACL reconstruction. Sports Health. 2024;16:149–54. 36935576 10.1177/19417381231155120 PMC10732117

[35] Webster KE, McPherson AL, Hewett TE, Feller JA. Factors associated with a return to preinjury level of sport performance after anterior cruciate ligament reconstruction surgery. Am J Sports Med. 2019;47:2557–62. 31381373 10.1177/0363546519865537

