# Supplementary material for: Translation, cross-cultural adaptation, and validation of the Chinese version of the injury-psychological readiness to return to sport scale
Source: BMC Sports Sci Med Rehabil. 2025 Apr 9;17:76. doi: 10.1186/s13102-025-01127-0 (PMC11984135; doi:10.1186/s13102-025-01127-0)
Supplement: Supplementary file 1 — Supplementary Material 1 [file 13102_2025_1127_MOESM1_ESM.docx]

**Appendix 1**

**The Items of the I-PRRS Scale and the Corresponding Chinese translation**

| **Items** | **Chinese translation** |
| --- | --- |
| 1. My overall confidence in returning to sport is… | 1. 我重返运动的整体信心… |
| 2. I will not be bothered by the pain of my injury when I return to sport… | 2. 我重返运动时不会感到受伤部位的疼痛… |
| 3. I will give my 100% effort when I return to training or competition… | 3. 我重返训练或比赛后会付出100%的努力… |
| 4. After returning to sport, my attention will not be distracted by my injury… | 4. 重返运动后，我的专注力不会因为受伤部位而受到干扰… |
| 5. My injury has recovered to the extent that it meets the physical requirement for me to return to sport… | 5. 我受伤部位的恢复状况能够满足我重返运动时的需求… |
| 6. My confidence in my athletic skill level or athletic competencies is… | 6. 我对自己的运动技术水平或运动能力的信心是… |
